# The History of Anthrax Weaponization in the Soviet Union

**DOI:** 10.7759/cureus.36800

**Published:** 2023-03-28

**Authors:** Ioannis Nikolakakis, Spyros N Michaleas, George Panayiotakopoulos, Theodore G Papaioannou, Marianna Karamanou

**Affiliations:** 1 Department of History of Medicine and Medical Ethics, National and Kapodistrian University of Athens School of Medicine, Athens, GRC; 2 Department of Pharmacology, University of Patras, School of Medicine, Patra, GRC

**Keywords:** biopreparat, bioweapon, bacillus anthracis, sverdlovsk, biowarfare

## Abstract

In this paper, we reveal the anthrax weaponization in the Soviet Union and its impact on biowarfare research, technology, and public health that resulted in the development of the first Soviet Anthrax vaccine and the subsequent vaccination of animals and humans en masse. We assume that there are cases that a biowarfare technology was incorporated into the civilian industry and benefited public health. However, the legacy of bioweapons today still poses an asymmetric threat to public health and safety.

## Introduction and background

Anthrax is a zoonotic disease caused by Bacillus anthracis that, for many years, plagued our civilization by killing livestock and humans. The disease was first mentioned by the Roman poet Virgil (70 BC to 19 BC) in his famous poem Georgics, in which he accurately described the disease in animals [1]. Centuries later, in 1727, the French physician Nicolas Fournier (1700-1781) classified the disease as charbon spontané and charbon contagieux. Fournier speculated that charbon spontané was a summer disease contracted from working under the sun, while charbon contagieux was contracted from consuming meat or being in close contact with wool or hides [2]. Eventually, B. anthracis was discovered in 1875 by the German physician and one of the founders of microbiology, Robert Koch (1843-1910). Anthrax is a non-motile, gram-positive, aerobic spore-forming bacillus, though the spores do not form unless exposed to high concentrations of oxygen (i.e., air). The resistant spores can survive in the soil for up to 48 years. Both domesticated and wild animals, primarily grazing herbivores such as goats, sheep, cattle, horses, and swine, are susceptible to infection. Human infections result from contact with infected animals or contaminated animal products [3].

The disease has three major forms: cutaneous, intestinal, and inhalation [4,5]. Cutaneous anthrax is the most common form, entering the body mostly via abrasions. Its incubation period ranges from 1-12 days (usually 5-7) [6]. Most cutaneous anthrax lesions emerge on exposed body surfaces such as the face, neck, and arms, presented as a painless necrotic pruritic papule (in most cases), developing into a necrotic ulcer with a black eschar [7]. Usually, lymphadenopathy and, more commonly, extensive edema in the surrounding tissue are typical for cutaneous anthrax, possibly accompanied by headache, fever, and malaise. Cutaneous anthrax has a mortality rate of less than 1% with antibiotic treatment but 20% without antibiotics [8].

Intestinal and oropharyngeal anthrax results from the ingestion of undercooked, infected meat and lead to multiple ulcerations in the alimentary tract and, consequently, hemorrhages. The fatality rate ranges from 4% to 75% [9]. Inhalation anthrax results from the inhalation of spores in contaminated animal products and bioattacks. Inhalation anthrax bacilli deposit in the alveoli and, when phagocytized by macrophages, are transported to nearby lymph nodes, where they multiply and release toxins, which, in turn, might cause hemorrhagic mediastinitis that may lead to necrotizing pneumonia [10]. In this stage, bacteremia occurs and may even result in meningitis. Its incubation period ranges from 1 to 7 days. The disease consists of two phases: during the prodromal phase, the most common symptoms are non-specific with myalgia, fever, and malaise, lasting from 4 to 5 days. In that phase, if antibiotics are initiated, the outcome of the treatment is usually successful. However, without antibiotics, the disease progresses rapidly to the second stage, a fulminant bacteremia phase with severe dyspnea, hypoxemia, and shock leading to death [11].

## Review

Why anthrax

One of the Soviet Union's preferred bioweapons was anthrax inhalation, particularly after 1979, when it replaced other bioweapons and was mass-produced. It was preferred due to the undifferentiated symptoms resembling a cold or flu during the first days of infection. This leads to the assumption that a patient would delay a visit to a healthcare professional, who should be both trained and knowledgeable in biological warfare mechanisms to diagnose the disease. Consequently, the weaponization of anthrax as a biowarfare agent is ideal because of the common early symptoms but also because of the lethality of the disease in the second phase.

Timeline of anthrax as a bioweapon in the Soviet Union

The Soviet Union’s biowarfare program was mostly based on the preexisting Tsarist-Russian bacteriological program, which started in 1885 with the establishment of the first Russian bacteriological station [12]. The Soviet Union officially initiated its biological warfare program in 1928, but evidence seems to conclude that the program had unofficially started some years earlier [13].

British SIS intelligence reports from 1924 confirmed the use of anthrax shells (with a capacity of 2 liters/shell), bombs, and mortars against pigs, with ''favorable'' results. Contrary to plague, anthrax would last in the environment for 1-2 hours, forming spores immediately, granting the bacillus sufficient resilience to infect a vast number of people [12].

In 1925, the Red Army's Military-Chemical Directorate (Военно-химическое управление-VOKhIMU) was formed and directed by the chemist Yakov Moisseevich Fishman (1887-1962), who would later become a recognized architect of the Soviet biological warfare program in its infancy [14].

In 1926, a bacteriological laboratory controlled by the VOKhIMU in Moscow was established, focusing on anthrax production. The director of the laboratory was Dr. A. N. Ginsburg, who was also a chemist. Already in May 1926, the first report of a new anthrax strain with enhanced virulence was filed, resulting in a 100% increase in mortality. New anthrax (intestinal and pulmonary) was tested on sheep and goats that died in less than 48-72 hours [12]. Many reports followed one of them stating the new potential for anthrax to be loaded into bombs and artillery shells. In addition, a combination of chemical agents and anthrax was again applied to animals. Combined with mustard gas, anthrax could easily penetrate the skin, increasing its virulence. During an experiment on pigeons, two laboratory assistants became infected and died, demonstrating the lethality of weaponized anthrax. 

In 1930, a secret report by the well-known military officer and politician Kliment Voroshilov (1881-1969) stated the possibility of German intentions of establishing bioweapon facilities, specifically researching anthrax and glanders [12]. This increased the interest in creating bioweapons in large quantities, and the idea of creating a vaccine for pathogens that could be released in the event of a bio-attack emerged.

A year later, the OGPU (an organization predating the NKVD) established the Facility of Special Purpose (Byuro osobogo naznacheniya [BON]) in the town of Suzdal. Additional facilities were established, though many of them were operating, as an integral part of gulag prison systems [15], known as sharashkas or, as officially named, Experimental Design Bureau (Russian: Опытное конструкторское бюро, Opytnoe konstruktorskoe bûro [ОКБ]). Due to the notorious and unethical research in the biowarfare facilities and the conviction and death of many top scientists in Tsarist Russia, a limited number of people voluntarily enrolled. To facilitate the research, many scientists in the USSR were imprisoned and forced to work in specific fields of science [15].

The Suzdal sharashka at the time was operated by 19 convicted scientists, all of whom specialized in infectious diseases. The reports from this era concluded that the biological warfare program was mostly oriented toward defending against biowarfare attacks. Some claims supported the existence of organized human experimentation programs in the sharashkas and gulags by many accounts [12].

A change was noted in 1934 in a memorandum by Voroshilov to Joseph Stalin (1878-1953), stating the need to develop offensive biowarfare agents and exploit the abilities of anthrax and tularemia [12]. The aforementioned research in the field of biological warfare might have been tested during the Japanese invasion of China in 1937 [16]. Allegedly, Soviet agents and Chinese communist guerillas poisoned wells and used anthrax for bio-attacks against the Japanese to impede their progress. According to the allegations of Colonel Tomosada Masuda (1892-1952), one of the scientists of the Japanese Biological warfare program, Soviet spies had infiltrated behind the lines, spreading anthrax and cholera to the Japanese army. As reported, around 2.000 horses had died through anthrax sabotage. Masuda purported the capture of many Soviet spies with ampules of anthrax bacilli and cholera [16]. The report mentioned two attacks using anthrax and six attacks using cholera. Furthermore, Masuda's claims were supported by a translated document titled “Chinese employment of Chemical and Bacteriological Warfare Against the Japanese”, disclosing several biowarfare attacks from September 1937 to August 1939. Furthermore, at that time, the Orenburg Biological Warfare Institute managed to create bacterial anthrax spore tablets that could dissolve in water [12].

Anthrax sanitary technical institute’s vaccine

Meanwhile, the development of an anthrax vaccine for animals progressed during these years at the Veterinary-Bacteriological Institute (Veterinarno-Bacteriologicheskii Institut), and as reported in 1931-32, it managed to produce an anthrax vaccine and immunoglobulins.

Based on the previous research, the Sanitary Technical Institute facility in Vlasikha-STI (later relocated to Gorodomyla and during WW2 to Kirov) began making progress on the development of a human vaccine in 1935 [17]. Notably, a team led by Nikolay Nikolaevich Ginburg and Alexander Lazarevich Tamarin developed two avirulent nonencapsulated bacillus anthracis strains, STI-1 and GIEV-III, from virulent ones by cultivating anthrax in coagulated horse serum [12]. Subsequently, they tested the vaccine on more than 2 million farm animals with success, and until 1960, 140.000.000 million animals were vaccinated, reducing the cases of anthrax in cattle and humans 10-fold [12].

Eventually, this research later materialized in the creation of the live anthrax vaccine for humans. The STI had managed to enhance the avirulent strains by making them resistant to antibiotics, so healthcare professionals would be able to administer antibiotics and the vaccine to anthrax victims simultaneously [18]. In light of these discoveries, it could be assumed that the Soviet Union had also developed offensive anthrax with antibiotic-resistant strains. Nonetheless, more than 90.000 soldiers were inoculated during World War 2, and during the following years, the state proceeded to vaccinate 131.663 individuals in Bessarabia. The vaccine was officially licensed for broad use through scarification in 1953 and inoculation in 1959 [12]. The Soviet STI-anthrax vaccine was the first vaccine that was created in biological warfare facilities and a known case of biowarfare technology being used for the benefit of the civilian industry and public health.

The post-world war II road to modernization

In 1953, in the Kirov facility, which had become one of the largest at that time, a leak of anthrax into the sewer system occurred [18]. Even though the sewers were disinfected, the anthrax spores, due to their resilience, remained there for many years. In 1956, a new strain of anthrax with increased virulence was found in one of the Kirov rodents by Vladimir Sizov. The army ordered the immediate cultivation of this new strain, which eventually led to the end product of weaponized anthrax 836.

During these years, the biological warfare program became one of the major programs in the USSR. In 1973, the Main Microbiological Industry Agency established Biopreparat, an entirely new network of institutes and facilities dedicated to biowarfare, just a year after the USSR signed the "Biological and Toxin Weapons Production Treaty," which prohibited the use of bioweapons. In the Biopreparat, the major undertaking task was the program “Ferment” [18], which aimed to create a new generation of biological weapons, many of which could be fitted into ICBMs (Intercontinental Ballistic Missiles). Among others, anthrax was codenamed L4 [18].

The Sverdlovsk outbreak

In the Sverdlovsk facilities (present-day Yekaterinburg), a leak of anthrax occurred on April 2, 1979. During a change of a clogged filter in an exhaust pipeline needed for the drying process of aerosolized anthrax, the filter was not restored until the next shift. Many workers at a nearby ceramic plant became ill in the days that followed, and the local party official, at the time President Boris Yeltsin (1931-2007), ordered a disinfection [19]. The disinfection caused the anthrax dust that had already settled in the ground to spread, resulting in even more cases of anthrax. Most of the infected workers died within a week of the exposure, proving the lethality of anthrax 836. The official death toll that was later announced was 66 people out of the 96 cases [19], but considering the destruction of the hospital files by the KGB, there is not a conclusive number of deaths or cases hospitalized. As a result, the death toll is thought to be higher and to have spread beyond Sverdlovsk. The KGB covered up the accident and attributed it to gastrointestinal anthrax originating from contaminated meat [13].

The rest of the world mounted suspicions about the cause of the accident but could not prove the origin of the disease [20]. Due to international pressure, a USA committee was accepted in 1987 to interview individuals that were implicated in the accident at Sverdlovsk. The American Specialists accepted the Soviet explanations that fell apart after the collapse of the Soviet Union. A study published in 1994 in Science named “The Sverdlovsk anthrax outbreak of 1979” by the geneticist and molecular biologist Matthew Meselson [19] strongly supported the idea that the origins of the accident could be traced back to the Sverdlovsk biowarfare facility. The contamination was widespread, and cattle died even 50 kilometers away from the area, following the wind direction that day at Sverdlovsk. M. Meselson concluded that the cause was the inhalation of anthrax that originated from the biowarfare compound [20]. In mid-April, a voluntary anthrax immunization campaign commenced in the Yekaterinburg region using the STI vaccine for ages ranging from 18 to 55.

The anthrax superpower and the collapse of the Soviet Union

After the accident, plans were made to relocate the facility from Sverdlovsk to Stepnogorsk (in 1984, the facility was relocated), with the aim of mass producing anthrax 836, as stated by Chairman Leonid Brezhnev in a 1982 secret decree, naming the facility Scientific Experimental and Production Base (SNOPB) [18].

One of the most significant scientists of the Biopreparat program was Ken Alibek (1950-) (Colonel Kanatzhan Alibekov while in the USSR). He had experimented on numerous deadly organisms (i.e., Y. Pestis, Marburg Virus) and anthrax, increasing their virulence even more [13]. He was assigned as the head of the facility in 1983, and he embarked on a mission to optimize anthrax dust production. In 1987, he managed to create a daily production of anthrax 836, amounting to 2 tons per day in this facility [13], while the annual anthrax production of the Soviet Union was 5.000 tons [13]. Alibek stated that, in optimal atmospheric conditions, 100 kg of anthrax could kill 3.000.000 people, and in the case of total war, one warhead of an SS-18 type ICBM fitted with an anthrax warhead could wipe out entirely the population of New York [13]. When the biological warfare program had reached the highest productivity in its history, the Soviet Union collapsed, leading many scientists (Ken Alibek among them) of the program to defect to the USA, providing valuable knowledge and insights into the covert biological warfare operations of the Soviet Union.

Dealing with the threat of an anthrax bioattack

**Figure 1:**
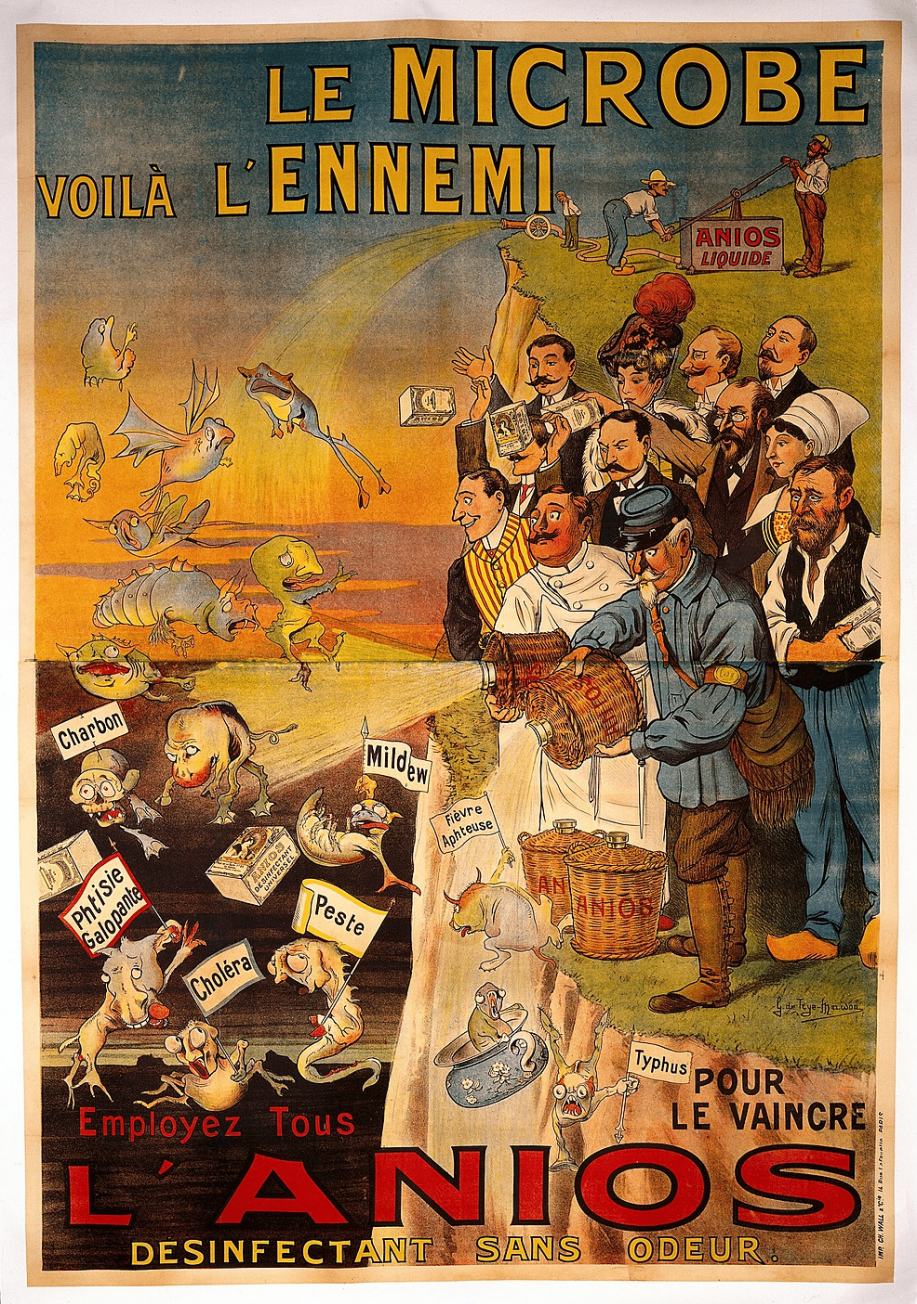
People using Anios disinfectant to destroy microbes representing infectious diseases. Color lithograph by G. de Trye-Maison, ca. 1910 Source: Wellcome Library, London. Free to use with attribution Attribution 4.0 International (CC BY 4.0).

Due to the high mortality rate of anthrax biowarfare strains, as mentioned above, it is crucial to highlight some characteristics of early recognition of anthrax bioattack and the appropriate management. In terms of biological warfare, CDC has classified *B. anthracis* as a category A organism of concern. An attack with *B. anthracis* would be the case of aerosolized exposure; thus Post-Exposure Prophylaxis (PEP) is recommended, suggesting immediate vaccination and antimicrobial therapy (Ciprofloxacin and Doxycycline are first-line options) [21]. When alternative preventive therapies are not available, monoclonal antibodies (raxibacumab and obiltoxaximab) can be used for treatment [22,23]. Both antibodies are held in the United States Strategic National Stockpile [24]. On a state level, the most important aspect of biodefense would be considered, the early recognition of an attack during the prodromal phase of infection, administering PEP, and therefore minimizing casualties. Figure 1 shows an illustration of people using anios disinfectant to destroy microbes representing infectious diseases.

## Conclusions

The Soviet Union regarded its own biological warfare program as of major importance, even though it was never employed due to its destructive power, leading to many medical discoveries that were later also used for public health and the increase of living standards in the USSR. During the last Cold War years, the Soviet Union was undoubtedly a biowarfare superpower due to the massive capability of anthrax weaponization. Biopreparat laid the foundation for Russia's largest producer of pharmaceuticals. The facilities were taken over by the nations formed after the fall of the Soviet Union, and along with them came the responsibility and dangers of having weaponized pathogens. The research on anthrax led to the vaccination of a large population of cattle and humans, reducing the incidence of anthrax dramatically, but also creating a weapon with immense destructive power that, if ever employed, would have unimaginable consequences. In the years following the collapse of the Soviet Union, the successor states that formed took control of these dangerous pathogens. The legacy of the development of anthrax as a bioweapon still poses a possible threat in the case of a future bioterrorist attack.
